# Nutrition and the Adaptation to Endurance Training

**DOI:** 10.1007/s40279-014-0146-1

**Published:** 2014-05-03

**Authors:** Keith Baar

**Affiliations:** Neurobiology, Physiology and Behavior, University of California Davis, 174 Briggs Hall, 1 Shields Avenue, Davis, CA 95616 USA

## Abstract

Maximizing metabolic stress at a given level of mechanical stress can improve the adaptive response to endurance training, decrease injury, and potentially improve performance. Calcium and metabolic stress, in the form of heat, decreases in the adenosine triphosphate/adenosine diphosphate ratio, glycogen depletion, caloric restriction, and oxidative stress, are the primary determinants of the adaptation to training. These stressors increase the activity and amount of peroxisome proliferator-activated receptor gamma coactivator 1 alpha (PGC-1α), a protein that can directly induce the primary adaptive responses to endurance exercise: mitochondrial biogenesis, angiogenesis, and increases in fat oxidation. The activity of PGC-1α is regulated by its charge (phosphorylation and acetylation), whereas its transcription is regulated by proteins that bind to myocyte enhancing factor 2, enhancer box, and cyclic adenosine monophosphate response element sites within the PGC-1α promoter. This brief review will describe what is known about the control of PGC-1α by these metabolic stressors. As the duration of calcium release and the amount of metabolic stress, and therefore the activation of PGC-1α, can be directly modulated by training and nutrition, a simple strategy can be generated to maximize the adaptive response to endurance training.

## Introduction

Every athlete knows that on race day he or she needs to be properly fueled to perform his or her best. However, when preparing for that day, does the same rule apply? As more is learnt about how the body responds to training it is becoming increasingly clear that in some instances it might be possible to get a better adaptive response if athletes are not fully fueled during certain training sessions. This brief review will discuss how nutrition can be used to maximize the adaptation to endurance training and how this can be used to promote healthy living in the general population and peak performance in elite athletes.

The goal for any endurance athlete is to maximize power/velocity at lactate threshold as this is the best determinant of endurance performance [1]. Lactate threshold is the point at which lactate accumulation in the blood shifts from a linear to an exponential relationship with exercise intensity, or as in the case of the aforementioned study a 1-mmol/L increase in lactate levels above baseline [1]. Lactate accumulation is the result of both increased production and decreased clearance [2]. The production of lactate increases for two primary reasons. The first is that epinephrine, calcium, and free adenosine diphosphate (ADP) and adenosine monophosphate (AMP) levels increase with exercise intensity. These factors activate glycogen phosphorylase, resulting in an increase in the breakdown of glycogen. The resulting rise in glucose 6-phosphate and fructose 6-phosphate, along with the increases in free ADP, activates phosphofructokinase and drives the glycolytic production of pyruvate. When the rate of pyruvate production outpaces the activity of pyruvate dehydrogenase, lactate is produced to regenerate nicotinamide adenine dinucleotide (NAD^+^). The second reason that lactate production increases is that as exercise intensity increases, larger motor units are recruited that tend to have fewer blood vessels and mitochondria [3]. Just as important as the increase in lactate production is the decrease in clearance that occurs with increasing exercise intensities. The decrease in clearance is largely the result of blood flow redistribution away from the liver and kidneys as epinephrine levels rise. As the liver and kidneys serve to convert lactate into glucose [4], when blood flow is shunted away lactate clearance will decrease. Therefore, the determinants of power/velocity at the lactate threshold are the sensitivity to epinephrine and the number of blood vessels and mitochondria within the muscle fibers of the largest motor units.

## The Muscular Adaptation to Endurance Exercise

From the perspective of a molecular biologist, maximizing mitochondria and blood vessels in fibers of the largest motor units is the role of peroxisome proliferator-activated receptor gamma coactivator 1 alpha (PGC-1α) and its binding partners. It has been known for over a decade that simply increasing PGC-1α can drive the formation of new mitochondria within a muscle [5]. More recently, PGC-1α has been shown to play a role in the control of fat oxidation and angiogenesis, suggesting that the adaptation to endurance exercise is mediated by PGC-1α. As PGC-1α is rapidly activated by endurance exercise [6, 7], this suggests that training should be designed to maximize PGC-1α activation. Naturally, this is an oversimplification. The regulation of mitochondrial mass is essential to organismal fitness and therefore redundancy has evolved to protect the organism from catastrophic failure. As a result of these redundant genes, muscles that lack PGC-1α are still able to increase mitochondria in response to exercise training [8]. However, without PGC-1α basal metabolic function is reduced because of a reduction in proteins of the electron transport chain and maximum aerobic capacity is dramatically reduced.

PGC-1α is a transcriptional coactivator, a protein that increases transcription without binding directly to DNA. Instead, PGC-1α interacts with transcription factors that bind to DNA in a sequence-specific manner. Therefore, the transcription factors identify the specific genes to turn on, whereas PGC-1α determines the volume. For example, by interacting with the nuclear response factors, PGC-1α can increase mitochondria [5]; by interacting with the peroxisome proliferator-activated receptors, PGC-1α increases fat oxidation proteins [9]; and by interacting with the estrogen-related receptor α, PGC-1α increases blood vessels [10]. Therefore in many ways, together with its binding partners, PGC-1α induces all of the muscular adaptations to endurance exercise.

If the key to future performance is the repeated activation of PGC-1α in training, the question becomes how can PGC-1α activity be maximized? PGC-1α is activated in two ways. First, existing PGC-1α protein can be modified either to make it go into the nucleus, where transcription takes place, or increase its ability to interact with its binding partners. There are two ways that it is known PGC-1α is modified: phosphorylation and acetylation [11, 12]. PGC-1α is most active when it is more phosphorylated and less acetylated. At the most basic level, phosphorylation is the addition of a negatively charged phosphate group to a protein. By a similar token, acetylation is the functional removal of a positive charge from a protein through the addition of a neutral acetyl group to a positively charged lysine residue. Therefore, PGC-1α is most active when it has more regions of positive and negative charges. The importance of the charge density on PGC-1α activity suggests that its translocation into the nucleus and its ability to interact with transcription factors is dependent on hydrogen bonding (the positive amino acids in one protein binding with the negative amino acids in another protein). Therefore, more phosphorylation and less acetylation equates to more negative and positive charges, better binding between PGC-1α and chaperone/transcription factors, and therefore higher transcriptional activity.

**Fig. 1:**
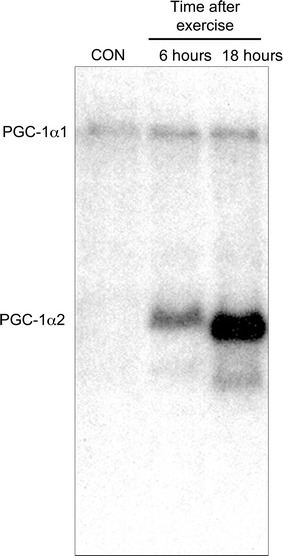
Endurance exercise increases the transcription of peroxisome proliferator-activated receptor gamma coactivator 1 alpha (PGC-1α) from both its canonical promoter (resulting in PGC-1α1 messenger RNA) and an alternative promoter (PGC-1α2) in control (CON) and exercised muscle. Note that PGC-1α2 is transcribed at high levels only 6 and 18 h after exercise (adapted from Baar et al. [6] with permission)

The second way to increase PGC-1α activity is to make more of the protein. The amount of PGC-1α protein is regulated transcriptionally through more than one promoter [13]. The canonical promoter is active ubiquitously, whereas the alternative promoter produces messenger RNA only in muscle and brown fat [10]. The gene products from the two promoters have been renamed to minimize confusion. Protein produced from the canonical promoter is now called PGC-1α1, whereas the protein from the alternative promoters are called PGC1α2-4 [10]. It was originally shown that following an acute bout of endurance exercise there was a rapid and profound increase in PGC-1α mRNA made from the alternative promoter (Fig. 1) [6]. The product of this alternative promoter is significantly shorter and is only expressed at high levels following exercise in both rodents [6] and humans [14]. The complex regulation of PGC-1α2 transcription by exercise has been elucidated over the past decade with the bulk of this work elegantly described in one paper by Akimoto et al. [15] and summarized below.

## Modulation of PGC-1α

From the data presented above, coaches and athletes with an eye on molecular biology would be looking to increase the charge and the transcription of PGC-1α2 in larger motor units to maximize endurance performance. The big question therefore is: how to increase the charge and the transcription of PGC-1α2? All of the research to date suggests that this is controlled by the metabolic intermediates: adenosine triphosphate (ATP) and its byproducts, phosphocreatine, NAD^+^, reactive oxygen species (ROS), cyclic adenosine monophosphate (cAMP), and calcium. The regulation of each of these intermediates will be discussed briefly below. For a more in-depth review of how each of these metabolic intermediates controls adaptation, please see the excellent recent series of reviews in the *American Journal of Physiology: Endocrinology and Metabolism* [16–21].

## PGC-1α Transcription

**Fig. 2:**
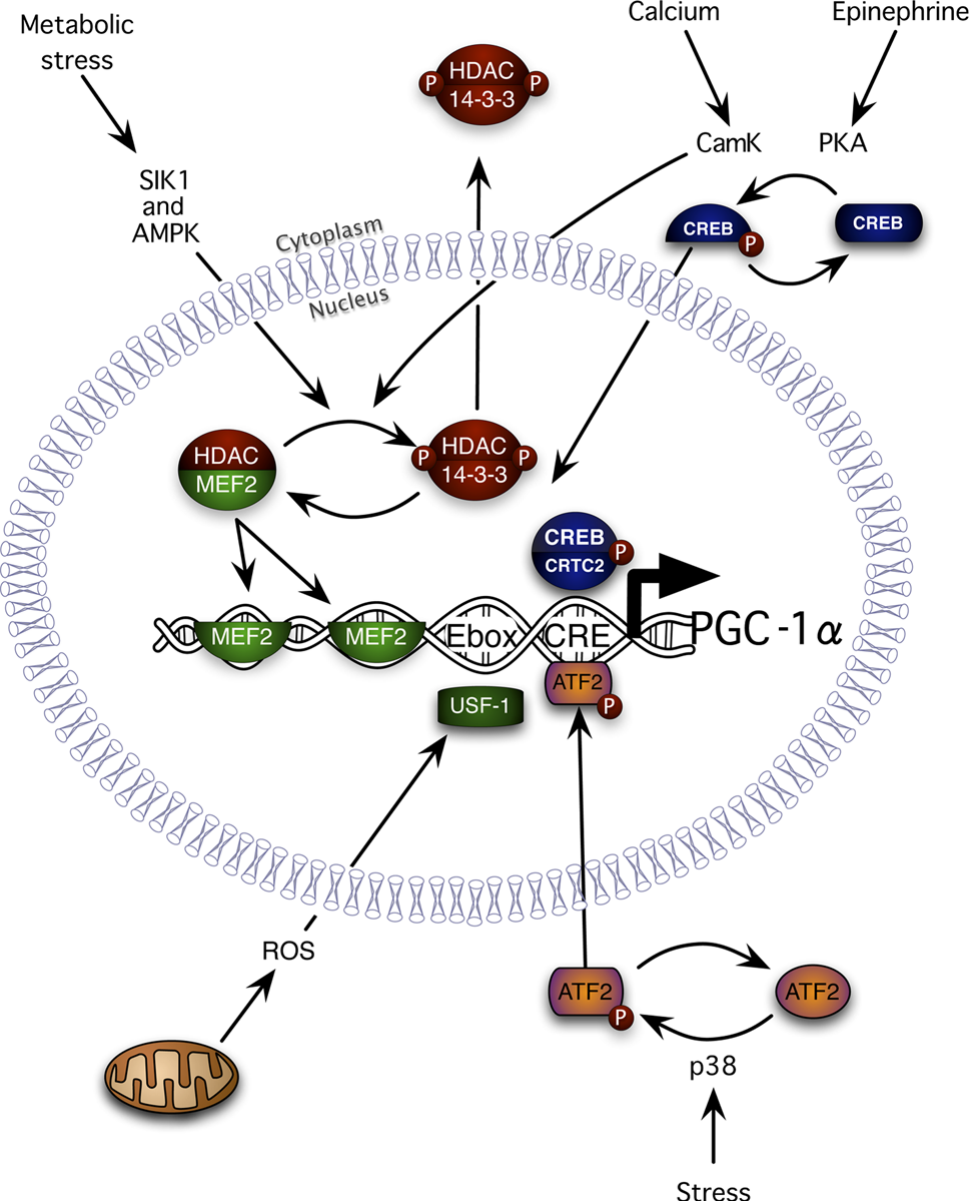
An illustration depicting the control of peroxisome proliferator-activated receptor gamma coactivator 1 alpha 2 (PGC-1α2) transcription. Exercise, reactive oxygen species (ROS), stress, and calcium release result in the activation of transcription factors that bind to the myocyte enhancer factor 2 (MEF2), enhancer box (Ebox), and cyclic adenosine monophosphate response element (CRE) regions of the PGC-1α alternative promoter and increase the production of PGC-1α2 mRNA. See text for complete details. *AMPK* adenosine monophosphate-activated protein kinase, *ATF2* activating transcription factor 2, *CamK* calcium/calmodulin kinase, *CREB* cyclic adenosine monophosphate response element binding protein, *CRTC2* CREB-regulated transcription coactivator 2, *HDAC* histone deacetylase, *PKA* protein kinase A, *p38* 38 kDa mitogen-activated protein kinase, *SIK1* salt-inducible kinase, *USF-1* upstream transcription factor

The components of the PGC-1α alternative promoter were first described in skeletal muscle by Akimoto et al. [15]. In that seminal paper, the authors ligated the PGC-1α alternative promoter to the firefly luciferase gene, electroporated the DNA into mouse tibialis anterior muscles, exercised the animals, and literally watched the transcription of the gene over time by detecting the light produced by luciferase. The authors then made point mutations in two regions of the promoter: one a cAMP response element (CRE) and the other two myocyte enhancing factor 2 (MEF2) binding sites (Fig. 2). As would be expected from the alternative promoter, the mutations did not change the baseline transcription of PGC-1α. However, either mutation completely prevented the activation of PGC-1α transcription by exercise [15]. Irrcher et al. [22] later extended this work showing a third important control region in the PGC-1α promoter: an enhancer box (Ebox) that is regulated by ROS. Therefore, the factors that control PGC-1α transcription are MEF2, a central Ebox, and proteins that bind to the CRE (Fig. 2).

## Calcium

Every time muscles are contracted, calcium is released from its intracellular store. While the majority of that calcium is used to initiate contraction, some of it leaks out of the myofibrillar space and activates a family of calcium binding proteins that are important in the adaptation to endurance training [23, 24]. One of these calcium binding proteins is an enzyme called calcium/calmodulin activated kinase II (CamKII). CamKII is a powerful activator of PGC-1α transcription and mitochondrial biogenesis. CamKII activates PGC-1α transcription through MEF2 and the cAMP response element binding protein. The CamKs activate MEF2 by releasing them from their inhibitors; the class II histone deacetylase (HDAC) proteins [25]. The CamKs do this by phosphorylating the class II HDACs and creating a binding site for the 14-3-3 chaperones (Fig. 2). When bound to 14-3-3, the class II HDACs are exported from the nucleus and degraded in the proteasome [26]. MEF2, which is no longer inhibited by the class II HDAC proteins, can then bind to the MEF2 elements in the PGC-1α promoter and activate transcription. CamK can also increase PGC-1α2 transcription through the CRE site by regulating the phosphorylation of the cAMP response element binding protein (CREB) [27]. Phosphorylated CREB can then bind to CREB-regulated transcription coactivator 2 [16] and together these proteins can increase PGC-1α transcription through the CRE site. As a result of controlling PGC-1α2 transcription, CamK overexpression induces mitochondrial biogenesis in vivo [28].

With prolonged contraction, the amount of calcium released for contraction decreases and the intracellular resting calcium increases [29]. However, intracellular calcium levels rapidly return to basal levels on the completion of exercise [30]. Therefore, the best way to increase the effects of calcium is to train a muscle fiber for a long time. This is the molecular rationale behind the idea of long slow training. The longer the athlete is on the bike, in the pool, or running, the longer calcium levels will be high in the muscle fibers and the longer PGC-1α transcription will remain elevated. When starting this type of long slow training, an athlete will first use their small motor units containing fibers rich in mitochondria. As they continue to work, glycogen in these fibers decreases and the larger motor units that contain fewer mitochondria have to be used. Therefore, near the end of a long training session an athlete has to recruit their larger motor units, and the calcium release in these fibers will provide the signal to increase mitochondria and blood vessels and improve power/velocity at the lactate threshold.

As described above, calcium increases PGC-1α transcription, but does not directly affect its charge state. The charge of PGC-1α (phosphorylation and acetylation) is regulated by stress and stress is highest when training is performed at high intensities. When training at high intensities, four things happen that affect PGC-1α activity: (1) phosphocreatine is depleted; (2) muscle glycogen is broken down; (3) lactate (and NAD^+^) increases; and (4) epinephrine levels rise. All of these provide signals that increase PGC-1α activity (see sections dedicated to each of these below).

## AMP-activated Protein Kinase

During high-intensity training, phosphocreatine is rapidly depleted from muscles [31]. To continue to train, ATP needs to be regenerated either through so-called anaerobic glycolysis or aerobic metabolism. In the process of breaking down and regenerating ATP, three other metabolites are made that affect PGC-1α activity: ADP, AMP, and creatine. As ADP, AMP, and creatine rise, this activates a protein called the AMP-activated protein kinase (AMPK; for an excellent recent review see Hardie et al. [32]). When inactive, AMPK binds to ATP and is readily dephosphorylated [33]. As ADP and AMP rise, during periods of high-intensity exercise, they bind to AMPK, allosterically activate it, and protect it from dephosphorylation [34]. The result is a highly active protein that can regulate both the charge of PGC-1α (phosphorylation) [35] and its transcription through the regulation of MEF2 [36]. AMPK regulates MEF2 in the same way as CamK, phosphorylating class II HDACs and inducing their release from MEF2 and removal from the nucleus by 14-3-3 [36, 37]. As AMPK and CamK have the same effects on PGC-1α2 transcription, from a molecular biology perspective it is not surprising that the metabolic adaptation to high-intensity interval training and long slow distance training are similar [38–40]. The high-intensity training turns on AMPK, whereas long slow distance work turns on calcium/CamKII signaling, resulting in PGC-1α activation and improved endurance.

## Glycogen

During exercise, glycogen stored within the muscle fibers is used to produce much of the energy needed. As the glycogen levels in the muscles fall, muscles sense the loss of glycogen and respond by activating AMPK and another important stress protein called the p38 mitogen-activated protein kinase (p38) [41]. Like AMPK, p38 increases both the charge of PGC-1α by phosphorylating it [12] and its transcription [42]. Puigserver et al. [12] showed that if they transfected cells with an activator of p38 (MKK6E) they could increase PGC-1α2 transcription. They then went on to discover three sites within the transcription factor binding domain of PGC-1α that were phosphorylated by p38. Mutating these three sites prevented the increase in PGC-1α2 and mitochondrial proteins induced by either MKK6E or cytokines that signal through p38 [12]. Transcriptionally, p38 activates the activating transcription factor 2 [43], which can bind to the CRE site and increase transcription (Fig. 2). In muscle cells, p38 is activated by endurance exercise [44] and this is potentiated in the glycogen-depleted state [45]. The activation of the gamma isoform of p38 is required for the increase in PGC-1α following exercise [42]. This indicates that p38, and by extension glycogen levels, are important regulators of the adaptation to endurance exercise.

## Nicotinamide Adenine Dinucleotide

Training above lactate threshold results in the accumulation of lactate within the working fibers. As discussed above, lactate rises within muscles as larger motor units with fewer mitochondria are recruited, and epinephrine, calcium, and free ADP/AMP increase, resulting in the activation of phosphorylase, glycogen breakdown, and accelerated glycolysis. The rise in lactate occurs as the redox state of the muscle changes and the ratio of NAD^+^/NADH increases [46]. NAD^+^ is required throughout metabolism as an electron carrier, but also serves another very important role: activating the NAD^+^-dependent deacetylases (for an outstanding recent review see White and Schenk [19]). Deacetylases are a family of proteins that remove acetyl groups from positively charged lysine residues in proteins, making them more positive. The best-studied member of the deacetylase family, sirtuin (SIRT1), is known to deacetylate and increase the charge of PGC-1α [11]. It was therefore initially quite surprising when it was found that PGC-1α acetylation and mitochondrial biogenesis following endurance exercise was normal in animals in which SIRT1 had been knocked out [47]. However, when the acetyltransferase, the enzyme that adds acetyl groups to the lysine residues in PGC-1α, was considered, the picture became clearer. Exercise not only activates SIRT1 but also inactivates at least one acetyltransferase, resulting in a dramatic decrease in the acetylation of PGC-1α following exercise [47].

SIRT1 is well known because it is activated by caloric restriction and in the long term is thought to increase the lifespan in lower organisms [48]. As it was thought that SIRT1 was activated by resveratrol, a component of the skins of the red grapes that appears in red wine, this formed the basis of some very interesting diets. Much to the chagrin of wine lovers everywhere, whether resveratrol directly activates SIRT1 or has beneficial effects on normal-weight individuals is still controversial [49–51]. However, SIRT1 is activated in muscle by caloric restriction and exercise and plays important metabolic roles. For instance, it is required for the positive effects of caloric restriction on muscle metabolism [52] and it can deacetylate and activate PGC-1α [11]. These facts together with the above data, suggest that to activate SIRT1 and therefore PGC-1α maximally, athletes should be encouraged to limit caloric intake before certain endurance training sessions and train at 75–100 % of maximum oxygen consumption.

## Epinephrine

As mentioned above, when athletes train at intensities above the lactate threshold a dramatic increase in epinephrine is seen [53]. Epinephrine also increases when athletes exercise for long periods without consuming carbohydrates or when they exercise in a glycogen-depleted state [54]. Epinephrine has many functions in the body that allow athletes to exercise at high intensities. It also plays an important role in the adaptation to exercise through the activation of PGC-1α2 transcription [10]. In mice, simply injecting a drug that mimics the effects of epinephrine increases the transcription of PGC-1α2, whereas mice lacking functional β-receptors do not experience an increase in PGC-1α2 transcription following exercise [55]. This suggests that epinephrine is important in the molecular adaptation to endurance exercise. Interestingly, β-agonists activate only the alternative promoter that is seen with exercise in muscle and brown fat [10, 56]. As with CamK, epinephrine does this by increasing CREB activity [56], this time through its second messengers cAMP and protein kinase A. The result of repeated intermittent rises in epinephrine is an increase in mitochondria and the formation of new blood vessels in muscle [10]. In contrast to this view, Robinson et al. [57] did not see an increase in PGC-1α expression or mitochondrial protein synthesis within the first 5 h after infusing the β-agonist isoproterenol. However, it is important to note that isoproterenol is not a specific β-agonist (it also activates α-adrenergic receptors and this can antagonize β-activation [58]). Therefore, whether catecholamines can acutely regulate PGC-1α in humans remains to be determined. However, the animal data are strong enough to encourage athletes to train in ways that increase epinephrine levels, such as in the heat [59], in glycogen depletion [54], or at high intensities [53].

## Reactive Oxygen Species

The last important factor in the control of PGC-1α is ROS. ROS, in the form of oxygen free radicals, are produced in the mitochondria as aerobic metabolism is performed. As athletes exercise, the rate of ROS production increases. Most ROS are quenched naturally by a series of cellular scavengers and antioxidants [60]. However, it seems that some ROS are necessary to increase the transcription of PGC-1α [22]. In fact, supplementation with high levels of synthetic antioxidants can blunt the normal increase in mitochondria with endurance training [61]. ROS activate PGC-1α2 transcription by modulating the activity of upstream stimulatory factor 1 (USF-1). USF-1 is a transcription factor that binds, in a ROS-dependent manner, to an Ebox in the first 850 base pairs of the PGC-1α2 promoter [22]. The requirement for this ROS-sensitive event in the transcription of PGC-1α2 supports the hypothesis that the consumption of high levels of synthetic antioxidants before training will blunt the training response [62]. However, in humans, 500 mg vitamin C and 400 IU vitamin E had no effect on endurance training adaptations [63]. Whether the higher levels of vitamins in many of the over-the-counter products are enough to scavenge the needed ROS during training has yet to be determined. The fact that moderate levels of vitamins C and E have no effect on adaptation does provide evidence that the lower naturally occurring levels of antioxidants present in fruits and vegetables would not have negative effects on PGC-1α-modulated training adaptations.

## Conclusion: Science-based Recommendations for Training to Maximize Endurance Adaptations

Using the molecular and metabolic information provided above, some simple nutritional strategies can be devised to make sure that athletes get the most out of endurance training. Once again, the goal with these recommendations is to maximize the mitochondrial adaptation to endurance exercise and improve performance at a later date. The goal of these suggestions is to maximize calcium, ADP/AMP, NAD^+^, cAMP, and ROS while minimizing injury. To do this, it is recommended:

- To start an “adaptive” session in a caloric deficit (i.e., in a fasted state such as in the morning before breakfast or in a glycogen-depleted state). This would make sure that SIRT1 activity would be high. It is important to remember that any time training is performed in a fasted or glycogen-depleted state, the perceived effort will be much higher and performance will decrease. This can be mitigated by carbohydrate mouth rinses and caffeine (see below).
- To consume a pre-training drink containing 200 mg of caffeine but free of carbohydrates and synthetic antioxidants. The caffeine serves primarily to decrease the perceived intensity of the training [64], but may also increase the release of calcium in the working muscles allowing greater activation of PGC-1α [24]. The absence of synthetic antioxidants promotes more mitochondrial biogenesis [62] because of improved PGC-1α2 transcription through USF-1 [22]. The lack of carbohydrate would maximize AMPK and p38 activity and epinephrine during training.
- Finally, that these adaptive training sessions should be performed at a low absolute intensity for a long time. This type of training would minimize the mechanical strain and keep calcium signaling high. However, beyond that, performing this training session in a fasted/glycogen depleted state would allow the athlete to benefit from a high SIRT1, AMPK, p38, and epinephrine state for the longest possible time, in theory resulting in the best possible adaptations.

It is important to remember that this nutritional strategy is designed to maximize the adaptive response to training and will decrease performance during training. Therefore, it is important to combine this type of “adaptive” session with more performance-based “quality” sessions. This is, in fact, what is common in many East African training schedules with a morning session before breakfast and a second higher quality session after lunch. Furthermore, many of these techniques were used by the three Canadians who competed in the marathon in the 2012 Olympics in London [65].

However, it is very important to remember that the job of a molecular biologist is to reduce complex processes to simple genetic models. In reality, of course, endurance performance is dependent on far more than PGC-1α. The nutritional strategy presented here uses the most recent scientific data to maximize the metabolic stress that leads to the increase in mitochondria and blood vessels that is required to increase power/velocity at the lactate threshold. However, this stress would also decrease immune function and therefore if used too often may increase infections and decrease training. Therefore, this type of “adaptive” training should simply be seen as another tool that can help an athlete build their endurance capacity. What the athlete does with high endurance capacity will depend on those high-quality training sessions that come when the athlete is completely fueled.
